# Supplementary material for: A yeast phenomic model for the influence of Warburg metabolism on genetic buffering of doxorubicin
Source: Cancer Metab. 2019 Oct 23;7:9. doi: 10.1186/s40170-019-0201-3 (PMC6806529; doi:10.1186/s40170-019-0201-3)
Supplement: Supplementary file 9 — Additional file 9. GO term-specific heatmaps for REMc/GTF-enriched clusters. GO term-specific heatmaps for significant GO process terms were generated as described in methods and Figs. 3 and 4. Any related child terms are presented in subsequent pages of the parent file name. GO terms with more than 100 children, with 2 or fewer genes annotated to the term, or a file size over 300KB are not shown. All heatmaps are generated with the same layout (see Figs. 3 and 4). [file 40170_2019_201_MOESM9_ESM.bz2 › Additional_File9_GOTermHeatmaps/Additional_File9_GOTermHeatmap/recombinational_repair.pdf]

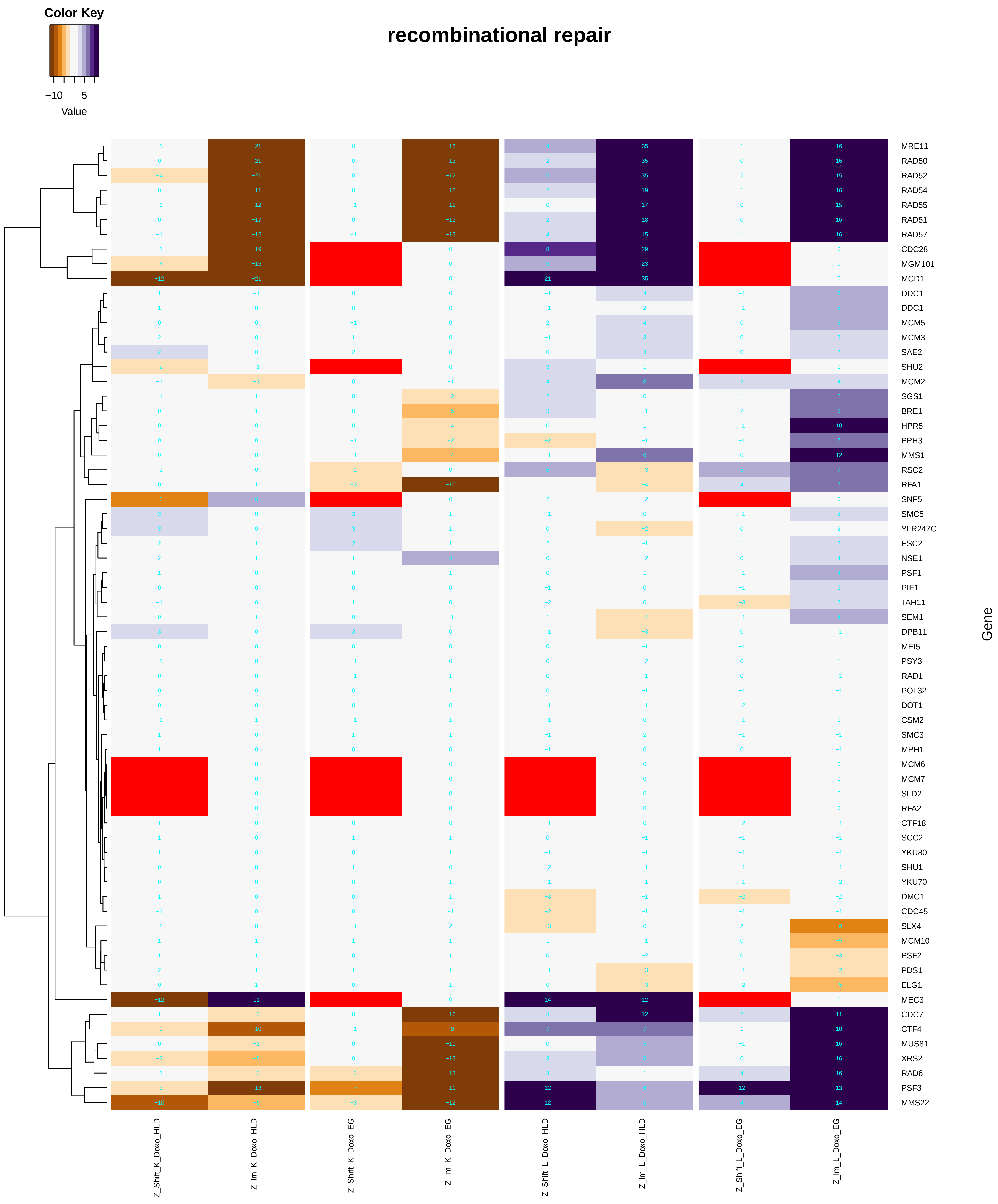
Color Key
−10
5
Value
recombinational repair
MRE11
RAD50
RAD52
RAD54
RAD55
RAD51
RAD57
CDC28
MGM101
MCD1
DDC1
DDC1
MCM5
MCM3
SAE2
SHU2
MCM2
SGS1
BRE1
HPR5
PPH3
MMS1
RSC2
RFA1
SNF5
SMC5
YLR247C
ESC2
NSE1
PSF1
PIF1
TAH11
SEM1
DPB11
MEI5
PSY3
RAD1
POL32
DOT1
CSM2
SMC3
MPH1
MCM6
MCM7
SLD2
RFA2
CTF18
SCC2
YKU80
SHU1
YKU70
DMC1
CDC45
SLX4
MCM10
PSF2
PDS1
ELG1
MEC3
CDC7
CTF4
MUS81
XRS2
RAD6
PSF3
MMS22
Gene
Z_Shift_K_Doxo_HLD
Z_lm_K_Doxo_HLD
Z_Shift_K_Doxo_EG
Z_lm_K_Doxo_EG
Z_Shift_L_Doxo_HLD
Z_lm_L_Doxo_HLD
Z_Shift_L_Doxo_EG
Z_lm_L_Doxo_EG

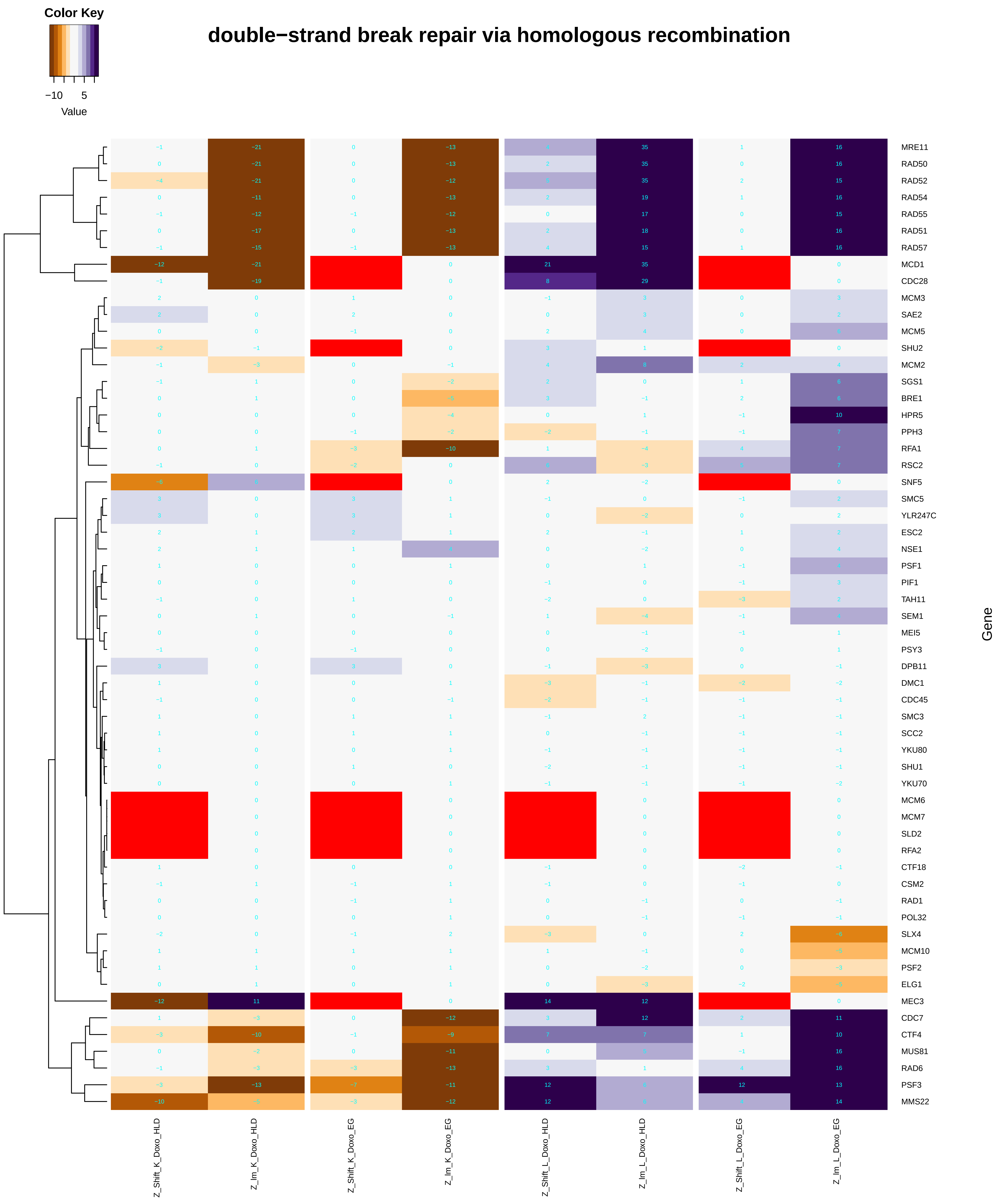
double−strand break repair via homologous recombination
Color Key
−10
5
Value
MRE11
RAD50
RAD52
RAD54
RAD55
RAD51
RAD57
MCD1
CDC28
MCM3
SAE2
MCM5
SHU2
MCM2
SGS1
BRE1
HPR5
PPH3
RFA1
RSC2
SNF5
SMC5
YLR247C
ESC2
NSE1
PSF1
PIF1
TAH11
SEM1
MEI5
PSY3
DPB11
DMC1
CDC45
SMC3
SCC2
YKU80
SHU1
YKU70
MCM6
MCM7
SLD2
RFA2
CTF18
CSM2
RAD1
POL32
SLX4
MCM10
PSF2
ELG1
MEC3
CDC7
CTF4
MUS81
RAD6
PSF3
MMS22
Gene
Z_Shift_K_Doxo_HLD
Z_Im_K_Doxo_HLD
Z_Shift_K_Doxo_EG
Z_Im_K_Doxo_EG
Z_Shift_L_Doxo_HLD
Z_Im_L_Doxo_HLD
Z_Shift_L_Doxo_EG
Z_Im_L_Doxo_EG

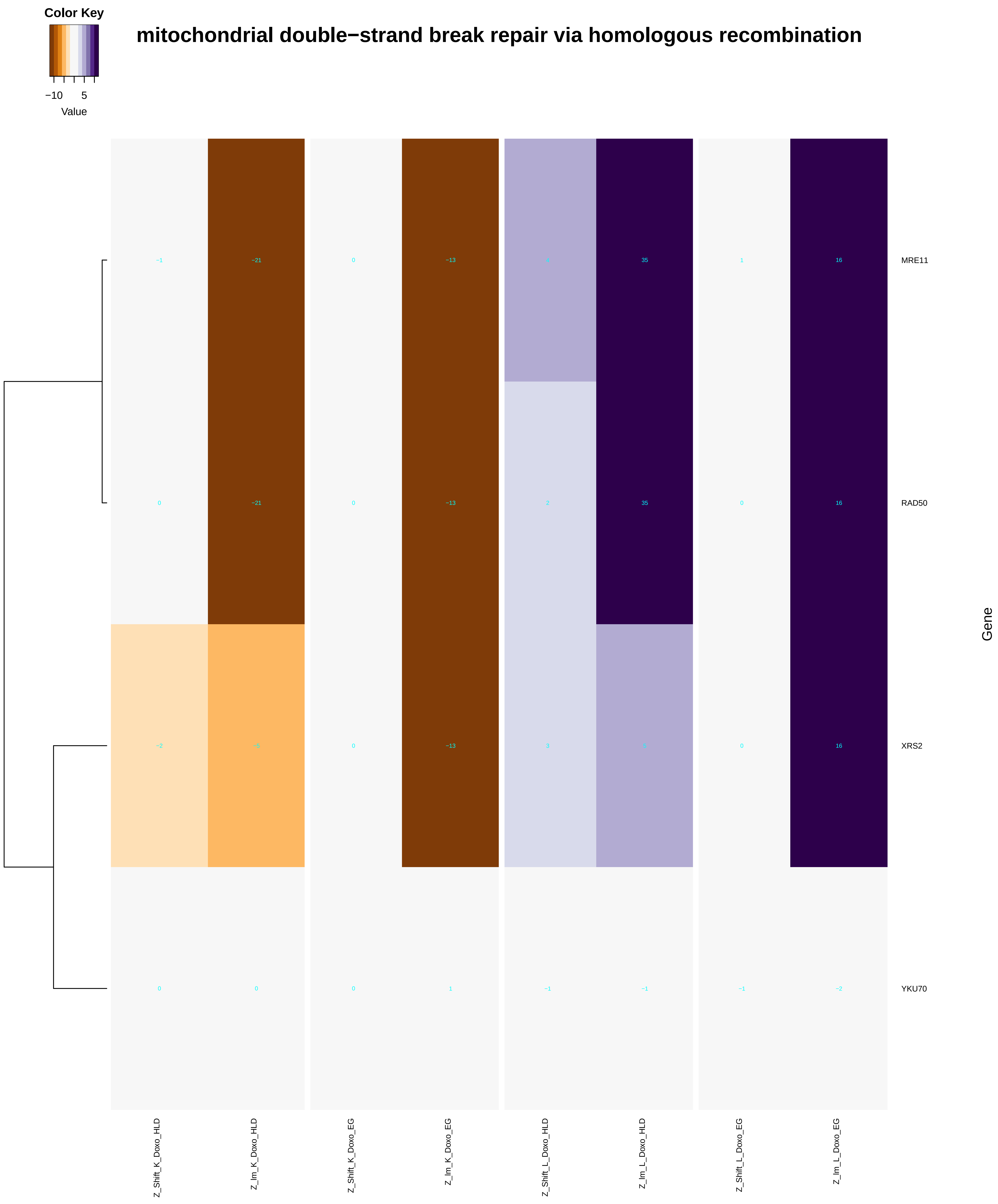

Color Key
−10
5
Value
mitochondrial double−strand break repair via homologous recombination
MRE11
RAD50
XRS2
YKU70
Gene
Z_Shift_K_Doxo_HLD
Z_Im_K_Doxo_HLD
Z_Shift_K_Doxo_EG
Z_Im_K_Doxo_EG
Z_Shift_L_Doxo_HLD
Z_Im_L_Doxo_HLD
Z_Shift_L_Doxo_EG
Z_Im_L_Doxo_EG

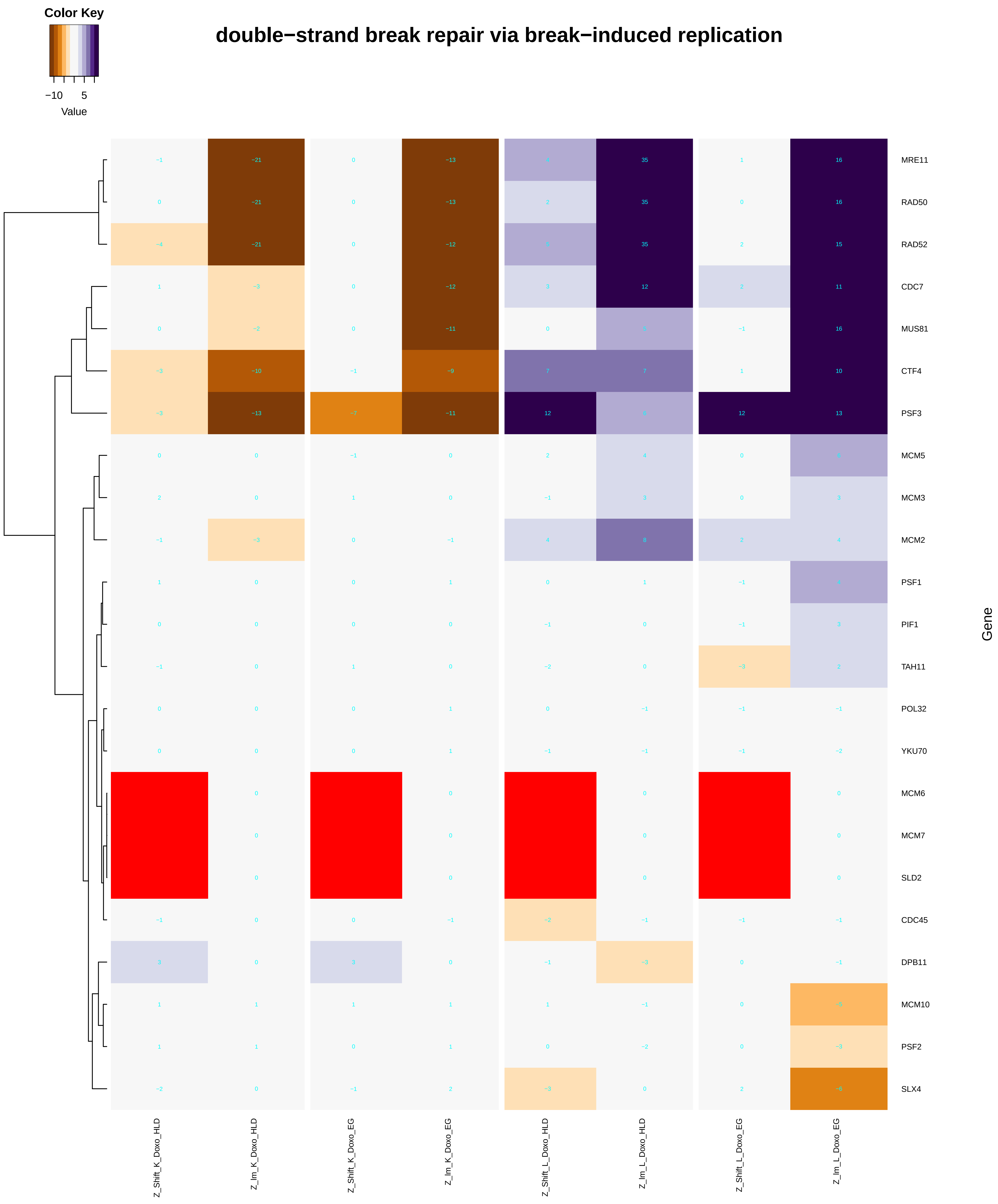

Color Key
−10
5
Value
double−strand break repair via break−induced replication
MRE11
RAD50
RAD52
CDC7
MUS81
CTF4
PSF3
MCM5
MCM3
MCM2
PSF1
PIF1
TAH11
POL32
YKU70
MCM6
MCM7
SLD2
CDC45
DPB11
MCM10
PSF2
SLX4
Gene
Z_Shift_K_Doxo_HLD
Z_lm_K_Doxo_HLD
Z_Shift_K_Doxo_EG
Z_lm_K_Doxo_EG
Z_Shift_L_Doxo_HLD
Z_lm_L_Doxo_HLD
Z_Shift_L_Doxo_EG
Z_lm_L_Doxo_EG

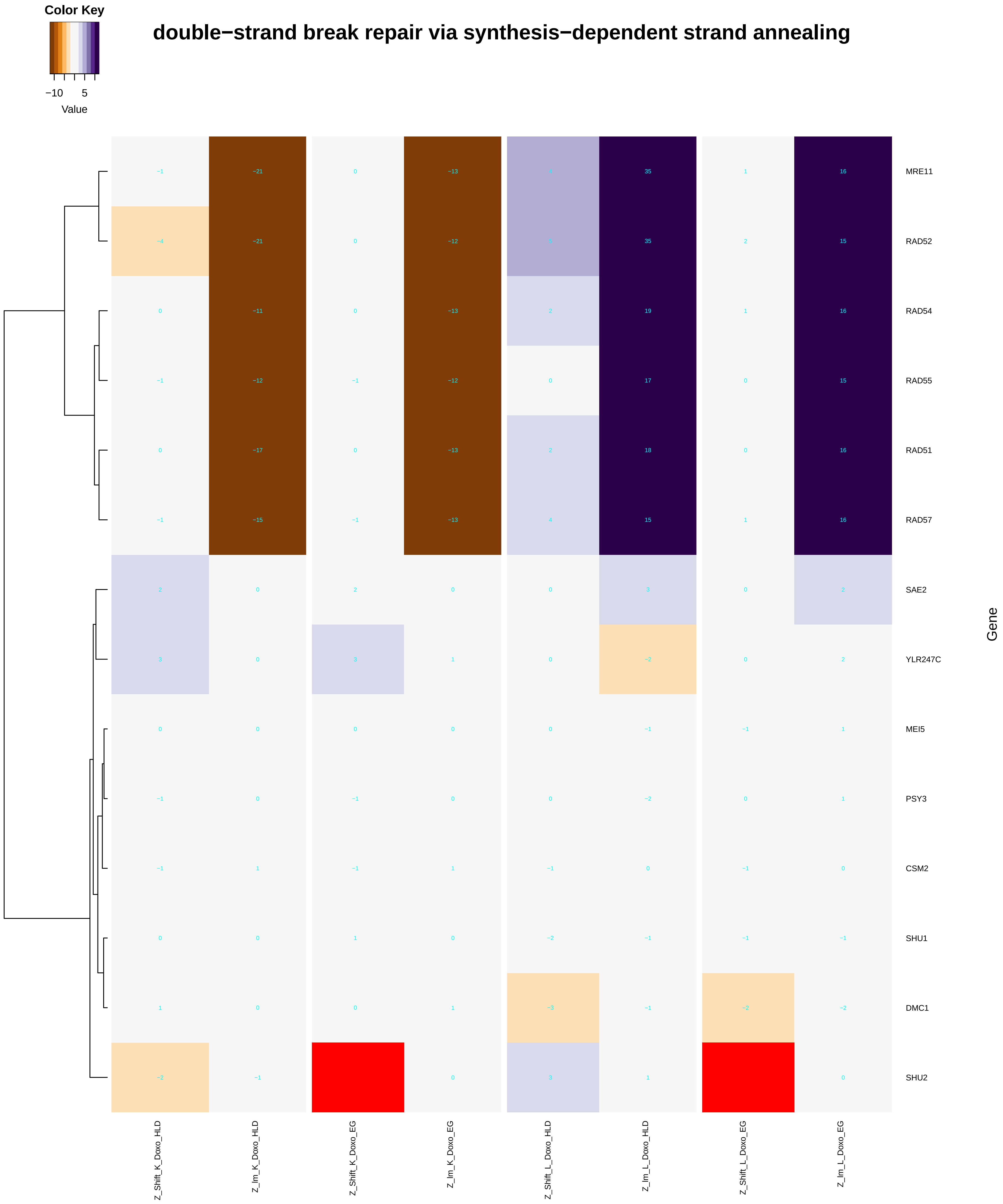
Color Key
−10
5
Value
double−strand break repair via synthesis−dependent strand annealing
MRE11
RAD52
RAD54
RAD55
RAD51
RAD57
SAE2
YLR247C
MEI5
PSY3
CSM2
SHU1
DMC1
SHU2
Gene
Z_Shift_K_Doxo_HLD
Z_Im_K_Doxo_HLD
Z_Shift_K_Doxo_EG
Z_Im_K_Doxo_EG
Z_Shift_L_Doxo_HLD
Z_Im_L_Doxo_HLD
Z_Shift_L_Doxo_EG
Z_Im_L_Doxo_EG

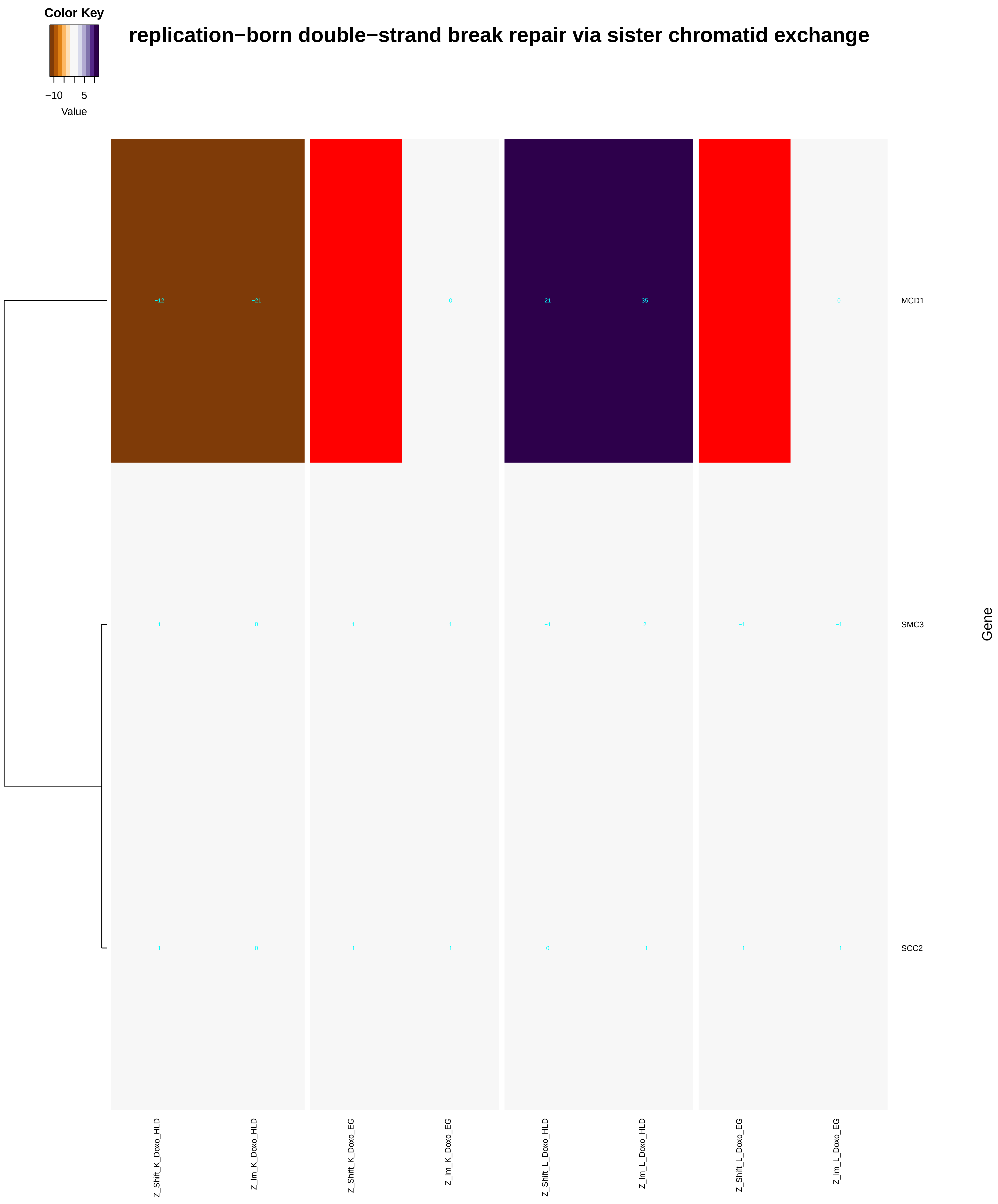

Color Key
−10
5
Value
replication−born double−strand break repair via sister chromatid exchange
−12
−21
0
21
35
0
1
0
1
1
−1
2
−1
−1
1
0
1
1
0
−1
−1
−1
MCD1
SMC3
SCC2
Gene
Z_Shift_K_Doxo_HLD
Z_Im_K_Doxo_HLD
Z_Shift_K_Doxo_EG
Z_Im_K_Doxo_EG
Z_Shift_L_Doxo_HLD
Z_Im_L_Doxo_HLD
Z_Shift_L_Doxo_EG
Z_Im_L_Doxo_EG
